# Supplementary material for: Splay-bend nematic phases of bent colloidal silica rods induced by polydispersity
Source: Nat Commun. 2022 Dec 1;13:7264. doi: 10.1038/s41467-022-34658-y (PMC9715686; doi:10.1038/s41467-022-34658-y)
Supplement: Supplementary file 1 — Supplementary Information [file 41467_2022_34658_MOESM1_ESM.pdf]

Supplementary Information: Splay-bend nematic phases of bent colloidal silica rods induced by polydispersity

Ramakrishna Kotni[1,†], Albert Grau-Carbonell[1,†], Massimiliano Chiappini[1,†], Marjolein Dijkstra[1,*], and Alfons van Blaaderen[1,*]

[1]Soft Condensed Matter, Debye Institute for Nanomaterials Science, Utrecht University, Princetonplein 1, Utrecht 3584 CC, The Netherlands
[†]Contributed equally to this work
[*]Corresponding authors: A.vanBlaaderen.uu.nl, M.Dijkstra@uu.nl

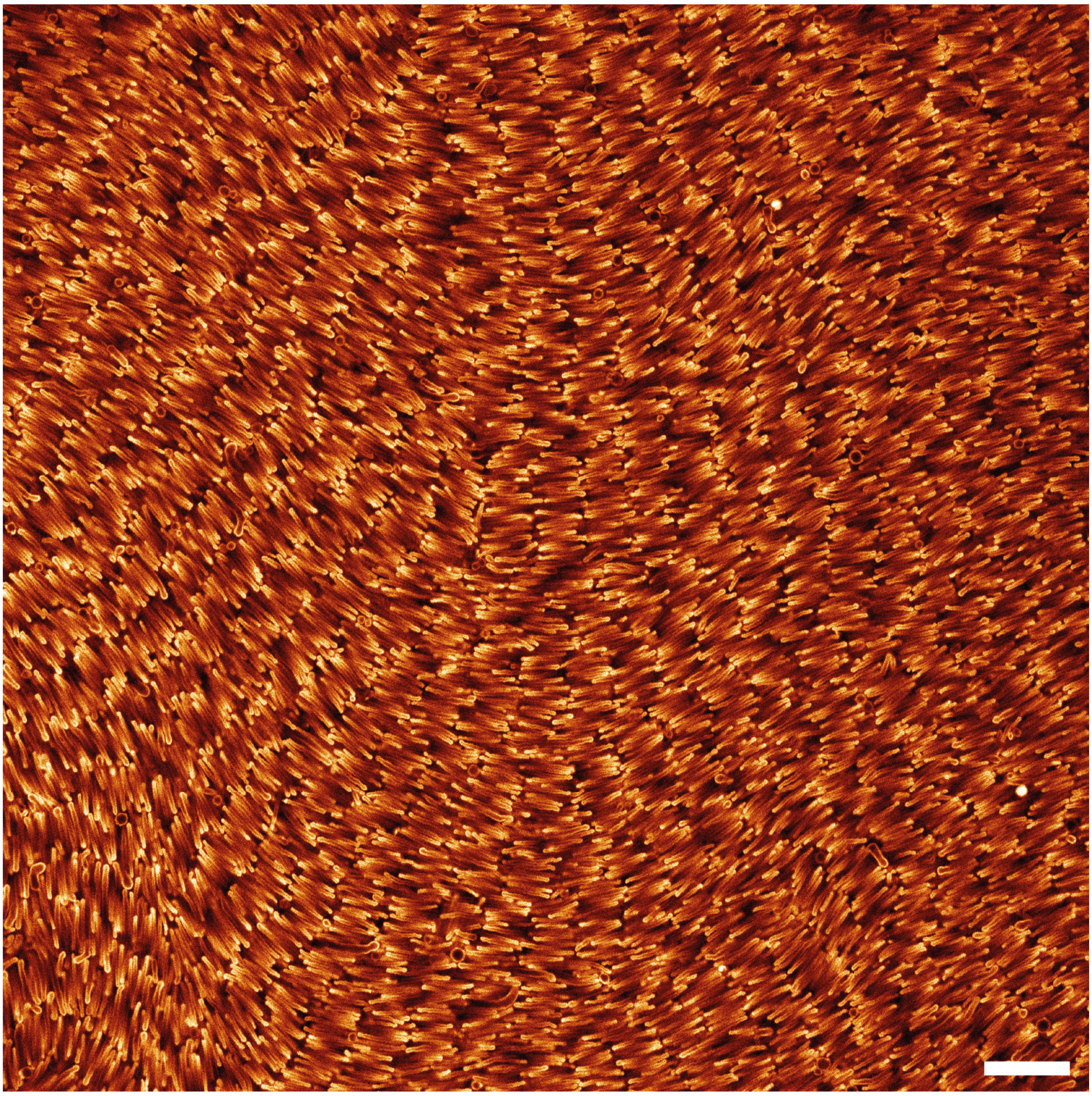

Supplementary Figure 1: N_{SB} phases of BSRs with particle dimensions of D= 490 ± 100 (20 %) nm, L_s = 2.110 ± 270 (13 %) nm, L_l = 3,190 ± 410 (13 %) nm) and α = 154 ± 8 (5 %) °.Scale bars 10 μm.

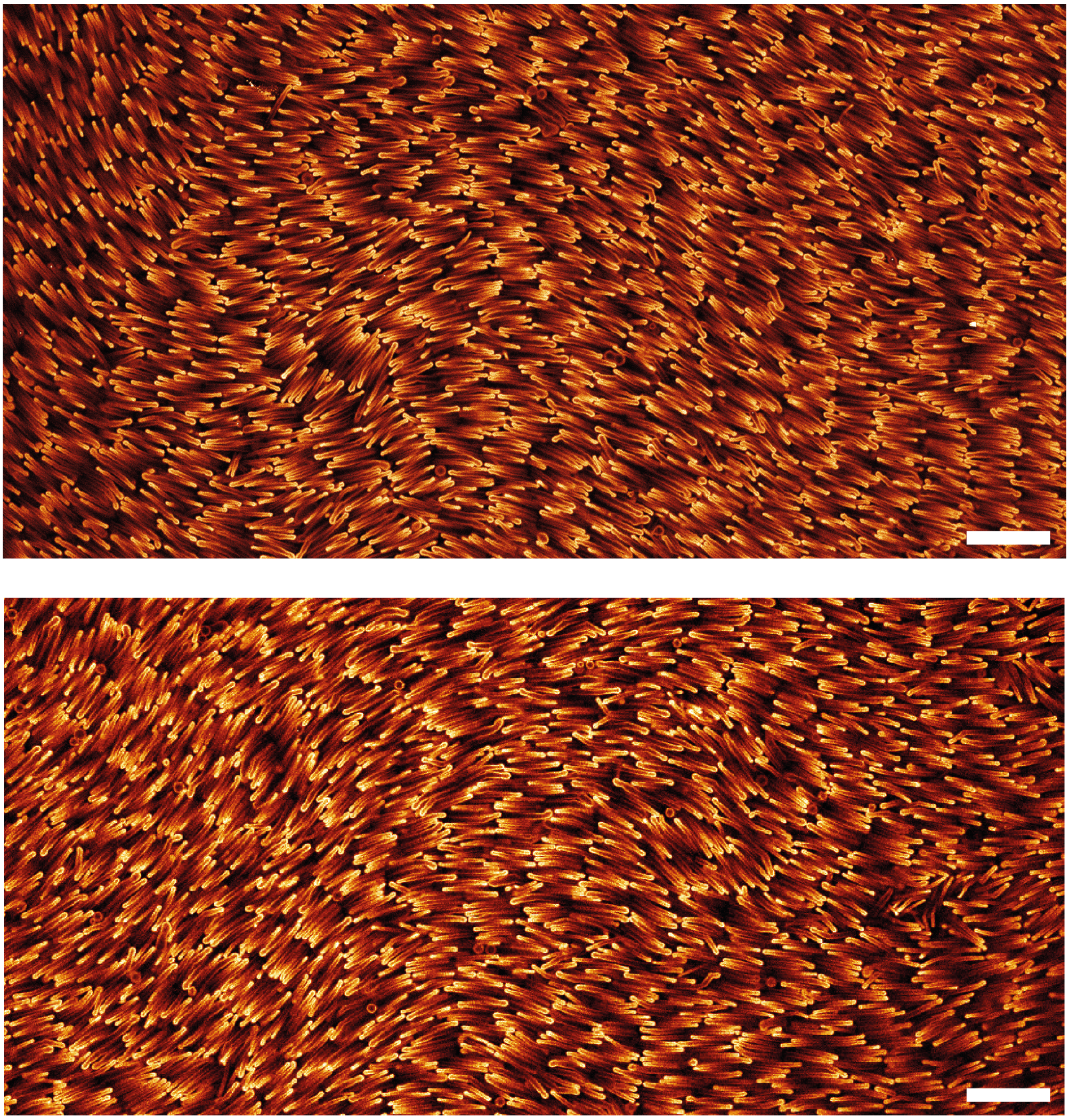

Supplementary Figure 2: N_{SB} phases of BSRs with particle dimensions of D= 490 ± 100 (20 %) nm, L_s = 2.110 ± 270 (13 %) nm, L_l = 3,190 ± 410 (13 %) nm) and α = 154 ± 8 (5 %) °.Scale bars 10 μm.

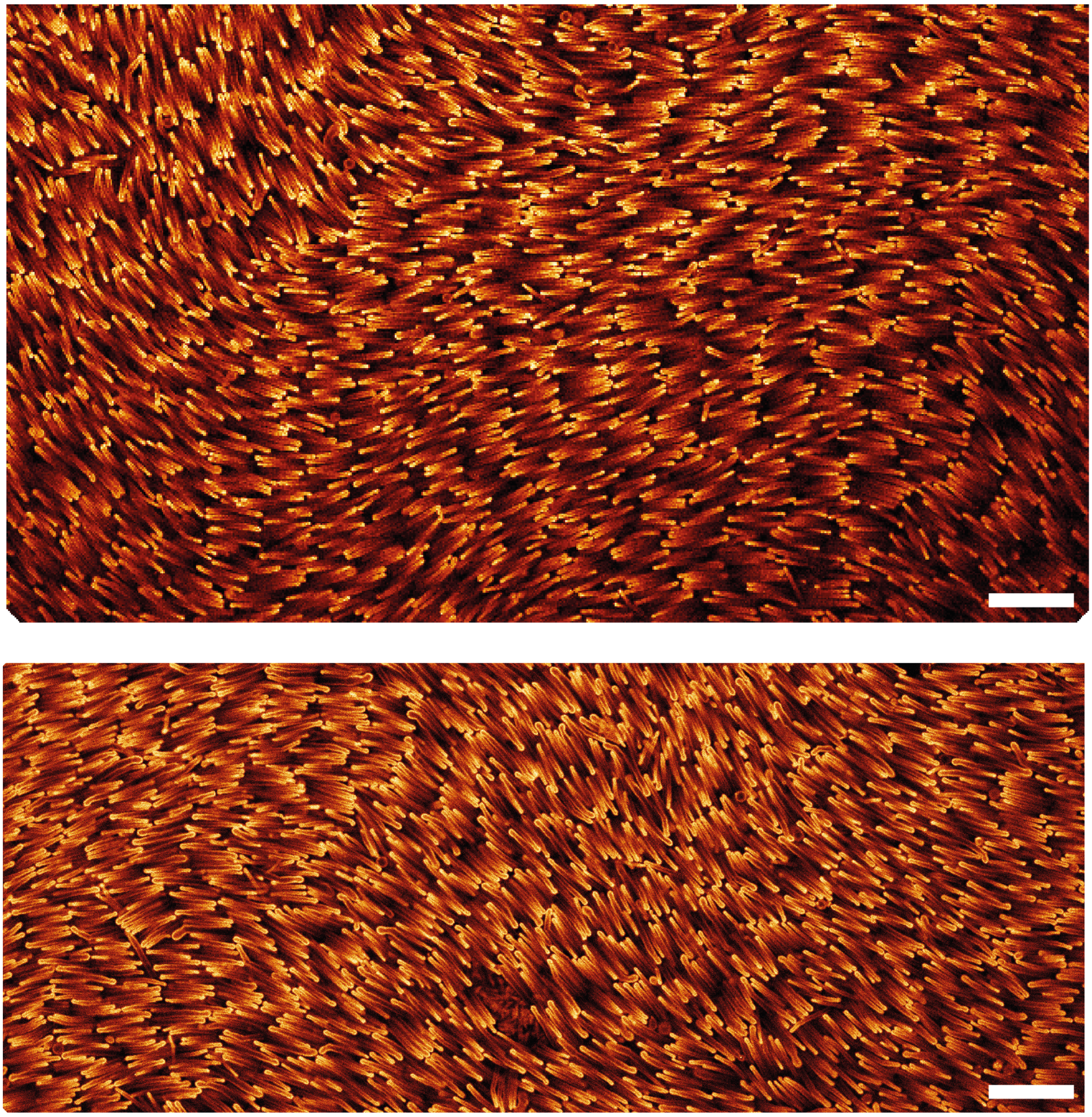

Supplementary Figure 3: N_{SB} phases of BSRs with particle dimensions of D= 490 ± 100 (20 %) nm, L_s = 2.110 ± 270 (13 %) nm, L_l = 3,190 ± 410 (13 %) nm) and α = 154 ± 8 (5 %) °.Scale bars 10 μm.

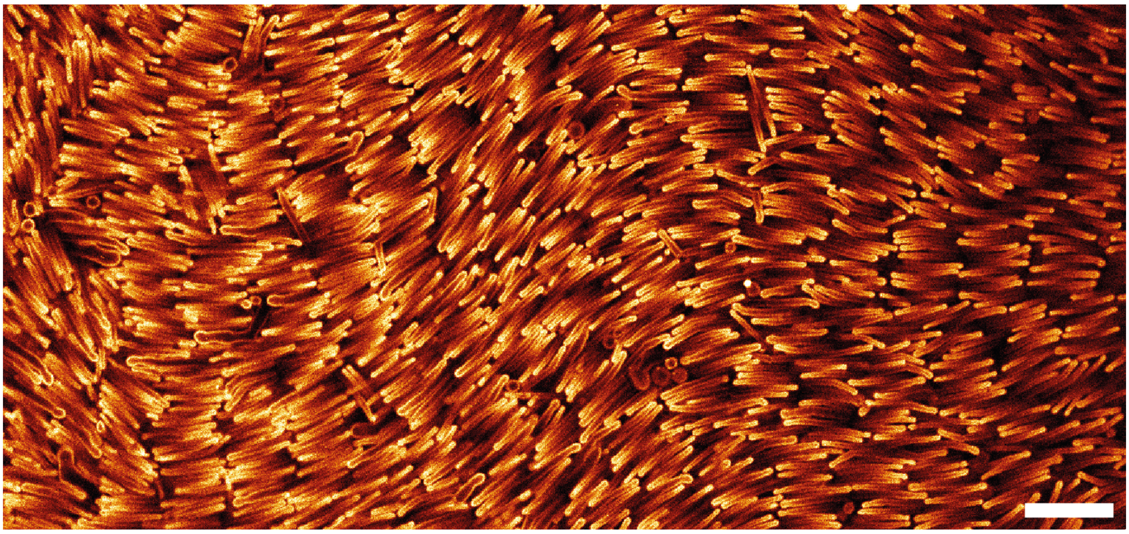

Supplementary Figure 4: N_{SB} phases of BSRs with particle dimensions of D= 490 ± 100 (20 %) nm, L_s = 2.110 ± 270 (13 %) nm, L_l = 3,190 ± 410 (13 %) nm) and α = 154 ± 8 (5 %) °. Scale bars 5 μm.

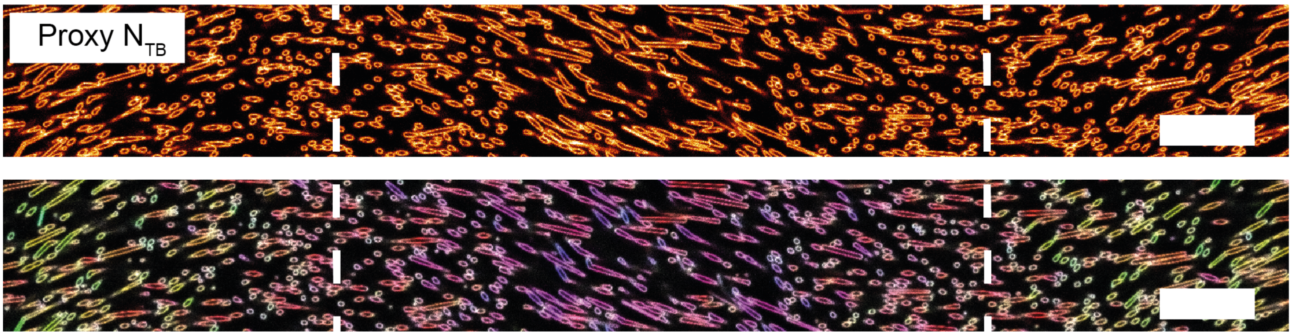

Supplementary Figure 5: Proxy, computationally generated fluorescence confocal microscopy image of a dispersion of rods with a N_{SB} configuration. The dashed lines indicate the regions where the nematic director becomes perpendicular to the imaging plane. Scale bars 10 μm.

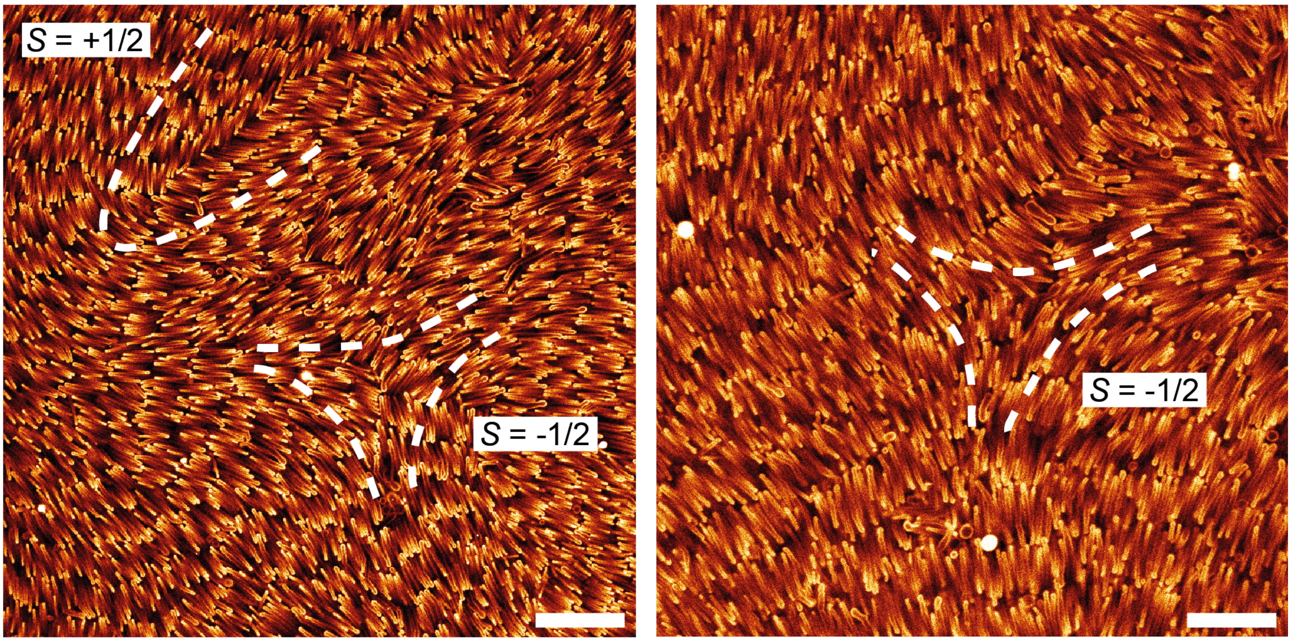

Supplementary Figure 6: Fluorescence confocal microscopy imaging of line defects in nematic phases of BSR with disclination strengths $S = \pm\ 1/2$.Scale bars 10 μm.

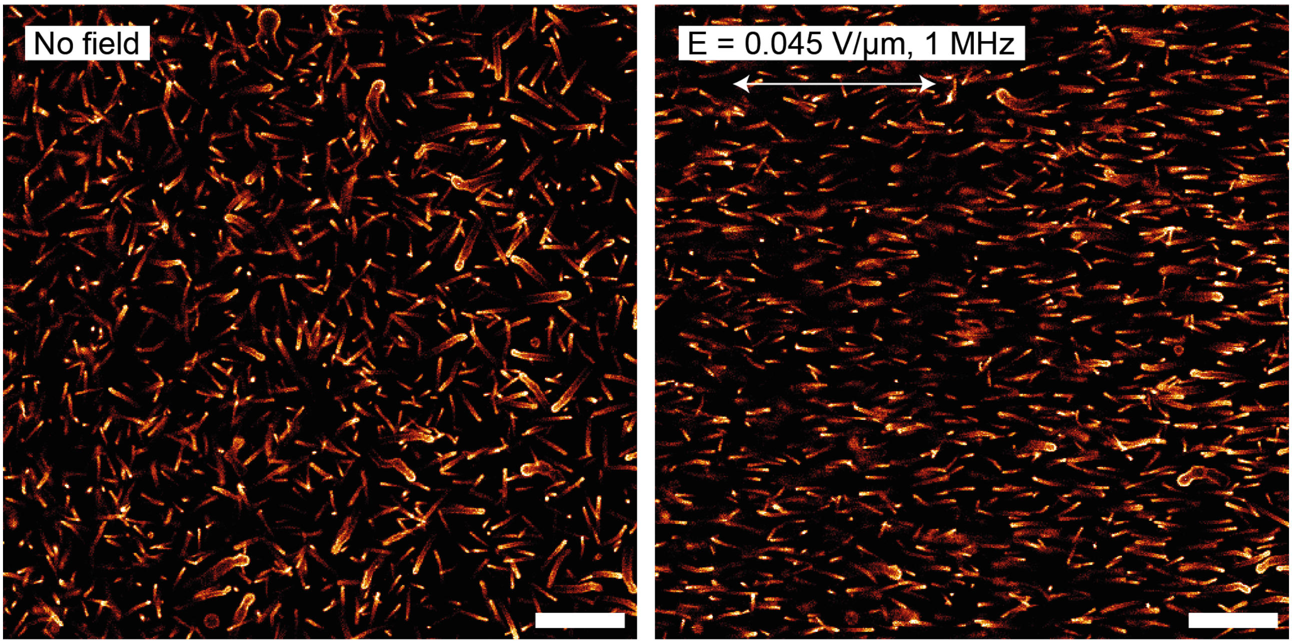

Supplementary Figure 7: Fluorescence confocal microscopy imaging of very diluted dispersions of BSRs. When an electric field (E = 0.045 V/μm, 1 MHz) was applied, the orientation of the particles aligned with the external field. Scale bars 10 μm.

Generation of confocal datasets of proxy N_{SB} and N_{TB} phases A 3D matrix of voxels containing the raw data of the proxy phases was created by the voxel-wise assignation of values to the voxels that would contain fluorophores for rods located at the previously obtained coordinates. The rods were assumed to have a fluorophore-rich shell, thus the voxels of the outermost parts of the rod were considered fluorescent. This dataset was considered as the ground truth, as it contained the unaltered information of the phase. This dataset was the processed with the Huygens Professional software to simulate confocal microscopy images. The dataset was convolved with theoretical point spread function of a confocal microscope, and the z-axis of the convolution was matched with that of the experimental data with respect of the modulation of the nematic field. Then photon noise was added to achieve realistic images with a signal-to-noise ratio of 10. This data sets were then analysed exactly as the experimental datasets.

Calculation of Debye length (κ^{-1}) This characteristic double-layer parameter was calculated for a monovalent electrolite in liquid medium following:

$$\kappa^{-1} = \sqrt{\frac{\epsilon_r \epsilon_0 k_B T}{2 N_A e^2 I}} \tag{1}$$

Where ϵ_r is the dielectric constant the solvent, ϵ_0 is the electrical permittivity of vacuum, k_B is Boltzmann's constant, N_A is Avogadro's number and e is the electron charge.
